# Optofluidic Fabry-Pérot Micro-Cavities Comprising Curved Surfaces for Homogeneous Liquid Refractometry—Design, Simulation, and Experimental Performance Assessment

**DOI:** 10.3390/mi7040062

**Published:** 2016-04-07

**Authors:** Noha Gaber, Yasser M. Sabry, Frédéric Marty, Tarik Bourouina

**Affiliations:** 1 Laboratoire Electronique, Systèmes de Communication et Microsystèmes, Université Paris-Est, ESIEE Paris, ESYCOM EA 2552, 93162 Noisy-le-Grand, France; frederic.marty@esiee.fr (F.M.); tarik.bourouina@esiee.fr (T.B.); 2 Center for Nanotechnology, Zewail City of Science and Technology, Sheikh Zayed District, 6th of October City, 12588 Giza, Egypt; 3 Electronics and Electrical Communication Engineering, Faculty of Engineering, Ain-Shams University, 1 Elsarayat St., Abbassia, Cairo 11517, Egypt; yasser.sabry@eng.asu.edu.eg

**Keywords:** Fabry-Pérot cavity, optical resonator, optofluidic sensor, on-chip refractometer, refractive index measurement, lab-on-a-chip

## Abstract

In the scope of miniaturized optical sensors for liquid refractometry, this work details the design, numerical simulation, and experimental characterization of a Fabry-Pérot resonator consisting of two deeply-etched silicon cylindrical mirrors with a micro-tube in between holding the liquid analyte under study. The curved surfaces of the tube and the cylindrical mirrors provide three-dimensional light confinement and enable achieving stability for the cavity illuminated by a Gaussian beam input. The resonant optofluidic cavity attains a high-quality factor (*Q*)—over 2800—which is necessary for a sensitive refractometer, not only by providing a sharp interference spectrum peak that enables accurate tracing of the peak wavelengths shifts, but also by providing steep side peaks, which enables detection of refractive index changes by power level variations when operating at a fixed wavelength. The latter method can achieve refractometry without the need for spectroscopy tools, provided certain criteria explained in the details are met. By experimentally measuring mixtures of acetone-toluene with different ratios, refractive index variations of 0.0005 < Δ*n* < 0.0022 could be detected, with sensitivity as high as 5500 μW/RIU.

## 1. Introduction

Refractometry is a well-known optical characterization technique to identify dielectric materials by measuring their refractive index (RI). It has various applications in chemistry, environmental science, material science, and even biology, as the RI of cells provides an important insight about its properties [1]. There are widely vast refractometry techniques depending on various optical phenomena such as beam refraction [2], light wave interference [3], optical cavity resonance of different configurations [4,5], and surface plasmon resonance [6]. Some of these techniques are matured and form the core of several products available in the market. Benchtop Abbe refractometers, for instance, are important in many labs whether they are optics labs, chemistry labs, or even material science labs. However, with the trend of miniaturizing devices, several on-chip versions of refractometers have emerged to provide cheaper and more compact devices that require small sample quantities. Many attempts has been made to integrate various refractometry techniques on chip, with each having their advantages and drawbacks. To ease the comparison, these techniques can be categorized under two major themes: “surface refractometry” and “volume refractometry” according to the amount of light and the nature of the light waves that pass through and interact with the sample material for sensing it. In surface refractometry, the sensing mechanism employs only the evanescent part of an electromagnetic wave to interact with the sample located at the surface of the resonator. Although these techniques can reach very high resolution of detecting RI change Δ*n* up to 9 × 10^−9^ RIU [7], they are generally at risk of being affected by surface contamination and not suitable for applications requiring thick surface penetration like measuring through big biological cells. For instance, the microtube ring resonators [8] can be used as optofluidic refractometric devices [9] with *Q*-factors up to several thousands [10] and sensitivities up to 880 nm/RIU for passive resonators [11], or even 5930 nm/RIU for an optofluidic tube coupled with a ring laser [12]. However, their evanescent field can interact with the surrounding environment at the distance of only few hundred nanometers inside and outside the microtube [11]. So imagine the application of water contamination detection as an example, the impurities will not be detected unless they accidentally swim beside the resonator surface through its small evanescent field area. On the contrary, in “volume refractometry” techniques, the light wave totally propagates through the sample renders the depth of interaction greatly increased. So, the sample can be entirely scanned by the detecting light beam when necessary. This comes at the expense of the attained sensitivity and resolution that is generally lower than the surface refractometry counterparts. Then it is useful to carefully choose the detection technique upon the priority required in the intended application.

Amongst the volume refractometry techniques to measure the RI of liquids, is the Fabry-Pérot (FP) optical resonator. It is simply a cavity of length (*d*) enclosed between two mirrors that causes the light to be multiply reflected between these mirrors. The length *d* is designed to provide constructive interference at certain wavelengths (λ), as it is equal to multiples of λ/2, leading to maximum signal at the output of the resonator. For other wavelengths, destructive interference with various degrees occurs, giving different signal levels with less values; hence, an interference spectrum is obtained for different incident wavelengths. By introducing the liquid sample inside the cavity, the peaks of the spectrum shift according to the RI of this liquid (*n*) and the corresponding effective cavity length (*n·d*), enabling the detection of the RI. The conventional detection method is done by recording the spectrum for at least one interference peak for the sample and for a reference liquid, then measure the shift between them. This method requires expensive equipment such as an optical spectrum analyzer or tunable laser source. An alternative method depending on the signal level variation has been introduced, first with ring resonators [3], then with FP resonators by our group [5]. The goal of this method is eliminating the need for such expensive spectrometry equipment, as only a photodetector is required to read the power change at a single wavelength. This can be achieved with enough sensitivity only if relatively high values of quality factors are attained by the resonator. For ordinary FP microfluidic refractometer with flat mirrors [13,14,15], *Q*-factor values are limited due to the light diffraction outside the cavity from the mirror boundaries as the mirror surface profile shape is not compatible with the Gaussian beam wave front′s curved shape. By employing curved mirrors and a micro-tube as in our device, the beam can be well confined leading to high *Q*-factors. Thereby, the interference peaks will have fast roll off enabling high sensitivity demonstrated by our previous work [5]. However, in our previous attempts the analytes used had different attenuation values at the employed wavelength range, which necessitates recording the spectra and normalizing them before tracing the power change with the RI. In this paper, we show performance with toluene and acetone mixture as both these liquids interestingly have the same attenuation values at our wavelength band, which enables studying the refractometer performance to investigate the possibility of spectrum-free detection. The experimental part is preceded by detailing the device design using a developed model and numerical simulation by HFSS (High Frequency Structural Simulator) that help the assessment of the refractometer performance.

## 2. Design

**Figure 1:**
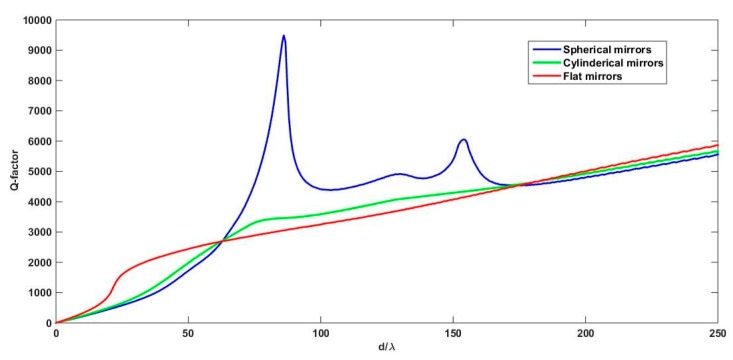
The theoretical values of the *Q*-factors for a FP cavity formed by straight mirrors, cylindrical mirrors, and spherical mirrors, plotted *versus* the diffraction length of the cavity.

For a highly sensitive refractometer, the FP resonators should provide sharp (or narrow) spectral lines to be more selective. That is represented by having a high *Q*-factor value at a specific wavelength. The *Q*-factor is given by:

(1) $$Q=F\frac{2nd}{\lambda }$$

where *F* is the finesse of the optical cavity that is defined as:

(2) F=2πN

where *N* is the number of round trips after which the energy bouncing inside the cavity drops to 1/e of its starting value. Hence, minimizing the energy losses inside the cavity is essential. The diffraction effect can be a major source of energy loss, as the energy escapes out of the open cavity one round trip after another. It can be overcome by making a proper design for the cavity mirrors resulting in a stable cavity. Long cavities and large mirror size are preferable for the high *Q*-factor they can produce, but this is obviously not compatible with miniaturization. Indeed, a cavity based on small mirrors imposes using small spot size for the light beams, which causes excessive beam expansion and light escapes the open cavity, when planar mirrors are used. That is why the *Q*-factor was limited in the previous attempts found in literature that implemented on-chip FP refractometer with flat mirrors [13,14,15]. On the other hand, earlier reports about FP cavities with spherical mirrors have demonstrated their excellent focusing capability. The curvature of the mirror focuses the beam in both directions perpendicular to the beam direction of propagation. Despite their high performances, the spherical resonators are difficult to miniaturize practically since the standard micro-fabrication technologies allow the realization of in-plane curved surfaces only. Thereby, as an intermediate solution, cylindrical mirrors can be implemented though the micro-fabrication process. These in-plane curved cylindrical silicon mirrors are adopted to achieve partial confinement in one lateral direction only. To evaluate the performance of a Fabry-Pérot cavity realized by different mirror shapes, a model for the theoretical *Q*-factor has been developed using Equations (1) and (2), where the corresponding round trip number *N* for the finesse calculation is deduced from the equation:

(3) $${\left|r_{m1}r_{m2}\right|}^{2N}\prod_{n=1}^{N}\eta_{n}=e^{-1}$$

where $r_{m1}$ and $r_{m2}$ are the field reflection coefficients of the cavity mirrors and $\eta_{n}$ is the round-trip coupling efficiency between the output field from the cavity and the fundamental mode of the optical fiber used for injecting the light into the cavity. The value of the finesse is limited by the coupling loss and the mirrors reflection, as *N* is calculated by solving Equation (3). Detailed analysis of the coupling efficiency can be found in references [16,17] where this modelling methodology was successfully applied in designing optical cavity based on planar mirror facing a three-dimensional curved mirror exhibition microscale size and curvature. In our case, we are using the in-plane curvature of the deeply-etched cylindrical mirrors and the out-of-plane curvature of the fluidic micro-tube to achieve three-dimensional control of the diffraction effect and arrive at a stable optical cavity. The *Q*-factor of the FP cavity is analyzed in Figure 1 for the 2-D confinement achieved by cylindrical mirrors, and 3-D confinement achieved by spherical mirrors and compared to the flat surfaces case, where there is no control on the diffraction effect. The Gaussian beam waist radius of the input/output lensed fibers used for light injection/collection is *w_o_* = 8 μm and the input/output fiber tip location is close to input/output mirrors while the beam waist location is one Rayleigh away from the fiber tip. The power reflectivity of the mirrors is assumed 97% and its radius of curvature is set by 140 μm. The *Q*-factor is plotted *versus* the optical diffraction length, given by the physical propagation length divided by the refractive index, normalized to the light wavelength.

**Figure 2:**
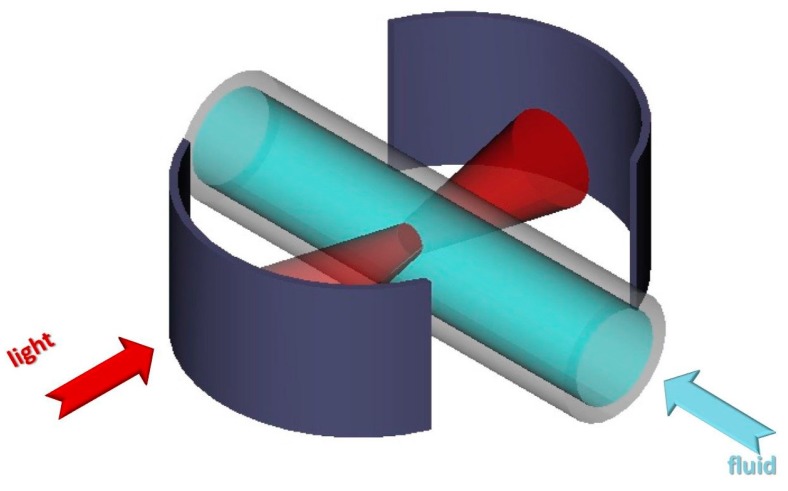
Schematic diagram of the cylindrical Fabry-Pérot cavity with the micro-tube inside.

As deduced from Figure 1, the performance of a cavity with cylindrical mirrors is slightly better than that with flat for intermediate cavity length. But with spherical mirrors, the *Q*-factor values are superior. But stile, such 3-D mirror curvature cannot be easily fabricated on chip. A workaround to overcome that is to decouple the 3-D curvature into two surfaces. One surface for the in-plan direction, which is the cylindrical mirrors, and a cylindrical rod lens laying on the chip provides confinement in the out-of-plane direction. The rod lens is formed by a micro-tube with the analyte inside, serves simultaneously for delivering the liquid under test and for light confinement. The schematic of the employed devise is shown in Figure 2.

**Figure 3:**
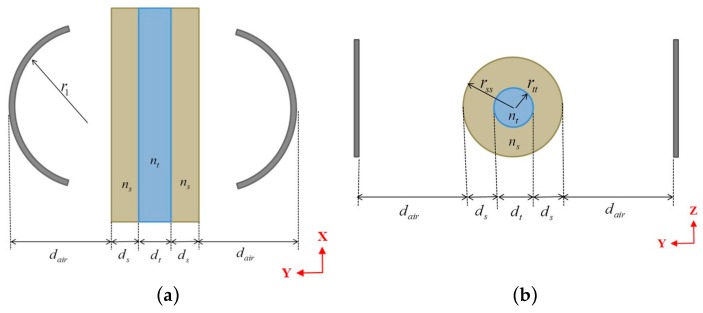
Schematic diagram for: (**a**) the horizontal cross section and (**b**) the vertical cross section, of the cylindrical Fabry-Pérot cavity with the micro tube inside indicating the design parameters and geometry.

For using the FP structure with the micro-tube in liquid analysis, the refractive index of the analyte may affect the cavity stability. Hence, the range of refractive indices that doesn′t degrade the performance much should be determined. The stability of the FP cavity can be investigated in a simple way by the ray matrix approach [18]. In this approach, each encountered surface is represented by a matrix, and then the equivalent matrix is obtained by multiplying them either symbolically or numerically by Matlab. Let the equivalent matrix components be A, and B, in the first raw; C, and D, in the second row. Then the stability condition is having the stability parameter (A + D)/2 be less than or equal to 1. We assume that the light behavior is decoupled in *XZ* (horizontal) and *YZ* (vertical) planes. Hence, each cross section is treated as a 1-D problem with schematics shown in Figure 3.

**Figure 4:**
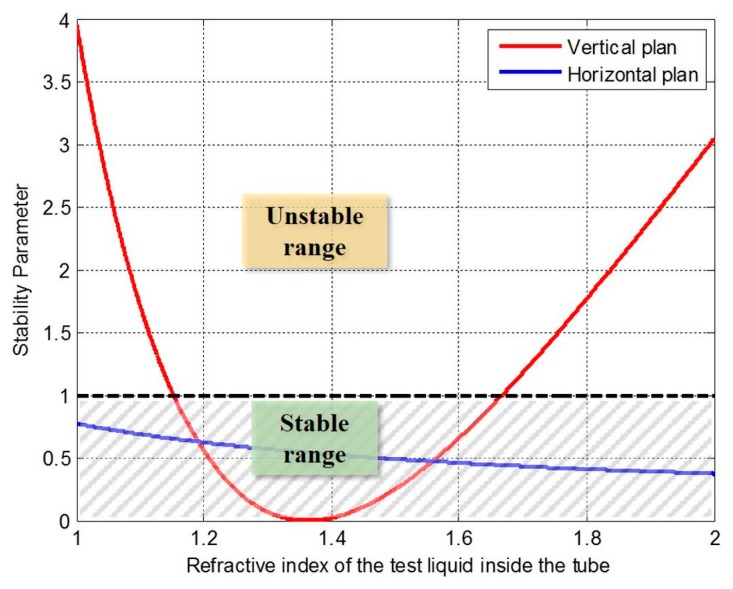
Stability parameter for different fluids inside the tube.

Thereby, we have two conditions that should be met simultaneously to achieve full stability in both directions. These conditions are:

(4) $$0\leq \frac{2}{r_{1}}\left(2d_{air}+\frac{2d_{s}}{n_{s}}+\frac{d_{t}}{n_{t}}\right)-\frac{4d_{air}}{r_{1}{}^{2}}\left(d_{air}+\frac{2d_{s}}{n_{s}}+\frac{d_{t}}{n_{t}}\right)-\frac{1}{r_{1}{}^{2}}\left({\left(\frac{d_{t}}{n_{t}}\right)}^{2}+4\frac{d_{s}}{n_{s}}\left(\frac{d_{s}}{n_{s}}+\frac{d_{t}}{n_{t}}\right)\right)\leq 1$$

(5) $$\begin{array}{cc} 0\leq & 4\frac{d_{air}{}^{2}}{n_{s}{}^{2}}{\left(\frac{n_{s}-n_{t}}{r_{tt}n_{t}}+\frac{1-n_{s}}{r_{ss}}\right)}^{2}+\frac{4}{r_{tt}}\frac{\left(n_{s}-n_{t}\right)}{n_{s}{}^{2}n_{t}}\left(4d_{air}+2r_{ss}-n_{s}\left(3d_{air}+r_{ss}\right)\right)\\ & +4\frac{d_{air}}{r_{ss}}\left(\frac{2}{n_{s}{}^{2}}-\frac{3}{n_{s}}+1\right)+4\frac{r_{ss}}{r_{tt}{}^{2}}{\left(\frac{n_{s}-n_{t}}{n_{s}{}^{2}n_{t}}\right)}^{2}\left(2d_{air}+r_{ss}\right)+4\frac{1-n_{s}}{n_{s}{}^{2}}+1\leq 1 \end{array}$$

where the geometrical parameters and refractive indices are shown in Figure 3. The parameters of the real device we have implemented are *d_air_* = 76 μm and *r*_1_ = 140 μm, and a fused silica capillary tube with dimensions of *d_t_* = 75 μm and *d_s_* = 26 μm. The stability may or may not be guaranteed according to the refractive index of the fluid inside the tube. Calculating the stability parameter for a range of refractive indexes from 1 to 2 to cover the condition of air and the majority of fluids that can be introduced inside the tube, as indicated in Figure 4, the stability is always assured in the horizontal plane. However, the vertical plane restricts it to the liquids whose refractive indexes are between 1.1526 and 1.6673. The proposed range of indexes constrains the applications of such device to some liquids only, which means gases are excluded as their refractive index is close to 1, if a high *Q*-factor is to be exploited.

## 3. Simulation

**Figure 5:**
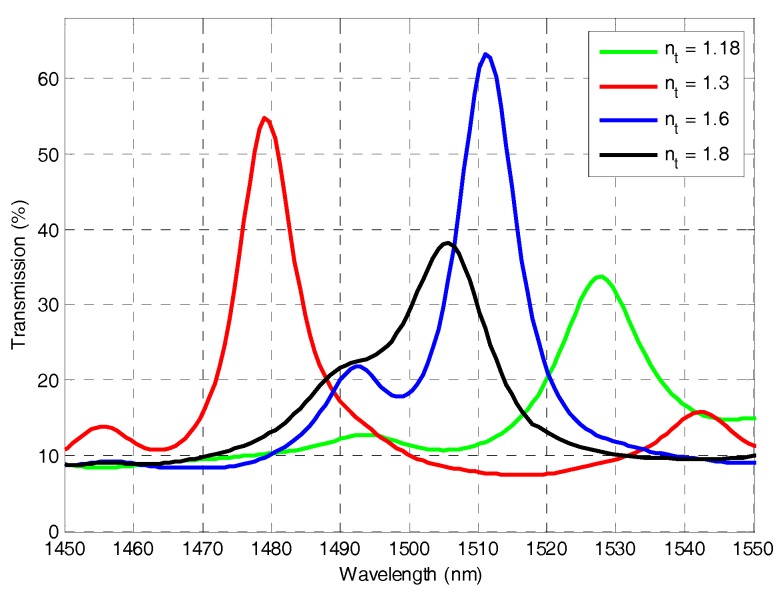
The transmission spectra of the curved cavity with a micro-tube filled with a test liquid of different refractive indices *n_t_*.

For accurate representation for the light wave, a cavity with scaled-down dimensions is simulated by HFSS program based on finite element method to have an idea of the electromagnetic modes behavior inside such cavities. If cavities with real dimensions were to be simulated, enormous calculation resources would be required. To overcome this problem, miniaturized versions of the cavities have been designed and simulated. Moreover, to render the simulation more efficient, we exploited the symmetries of the design in respect to the *XY* and the *YZ* planes to simulate only one quarter the cavity volume. For further simplification and size reduction, cavities with a single silicon Bragg layer per mirror have been simulated. Also, the thickness of the silicon layer is taken equal to 111.4 nm equivalent to only one quarter of the wavelength (in silicon) with respect to the reference central wavelength of 1550 nm in vacuum. The scaled-down cavity has geometrical parameters of 9.85 μm for the physical length, 7.5 μm for the radius of curvature, 6.25 μm for the micro-tube eternal diameter, and 0.75 μm for the internal diameter; the spot size of the exciting Gaussian beam is 0.9 μm. Checking the stability of such downscaled cavity, the range of the filling liquid *n_t_* that achieves stability in this case is between 1.15 and 2.03. Thereby we simulate a tube filed with different fluids that have different indices near the limits of that RI values range that achieves stability, lower and higher than the silica refractive index (the material of the walls of the tube). The selected values of the test fluid *n_t_* are 1.18, 1.3, 1.6, and 1.8, all within the stability range. The transmitted output power spectra for these cases are shown in Figure 5.

**Figure 6:**
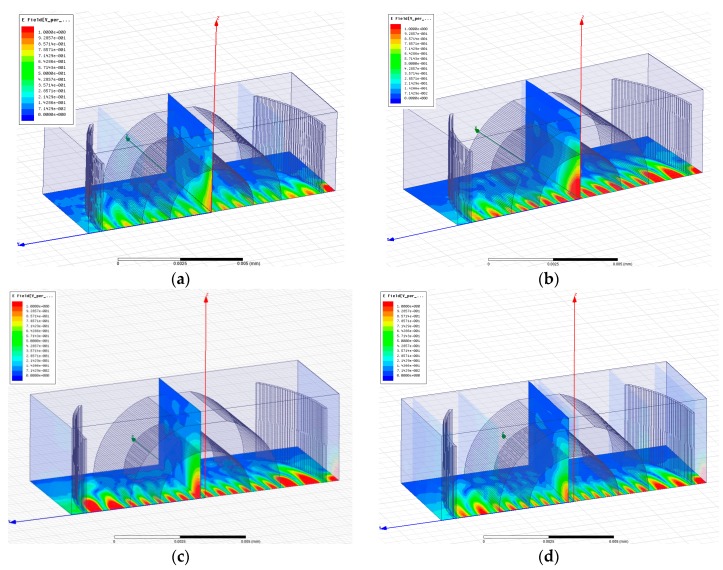
The electric field distribution at resonance for different test liquids (**a**) *n_t_* = 1.18, resonance at 1528 nm; (**b**) *n_t_* = 1.3, resonance at 1576 nm; (**c**) *n_t_* = 1.6, resonance at 1511 nm; (**d**) *n_t_* = 1.8, resonance at 1505.7 nm.

**Figure 7:**
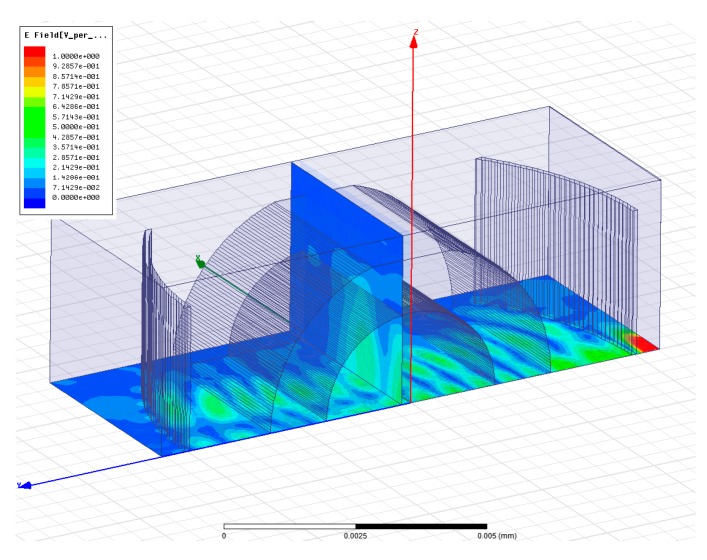
The electric field distribution of higher order resonance mode at 1542 nm for the test liquid *n_t_* = 1.3.

As noticed from the transmission spectra, there is a large peak for each case along with one or several smaller peaks. The largest one corresponds to the fundamental resonance mode as revealed form the field distributions shown in Figure 6. While the smaller peaks correspond to the higher order Hermite-Gaussian transverse modes, as noticed from the field distribution for the higher order resonance mode at 1542 nm for the test liquid *n_t_* = 1.3 shown in Figure 7. The cross sections have three spots in the transverse direction (note that only a quarter of the cavity is shown, hence we can see one and a half spot in *X*-direction, but it is mirrored around the *Z*-axis as we have even symmetry). These modes are typical resonance modes in FP resonators with spherical mirrors [19], and they appear also in FP cavities with cylindrical mirrors [20].

To investigate the field confinement quantitatively, Table 1 states the *Q*-factor values the confinement distances that is taken as the lateral distance from the maximum field value at the center of the spot to the value of half the maximum from the field distribution plotted Figure 6.

As theoretically predicted and as can be inherited from the field distributions in Figure 6, when the test fluid has refractive index less than that of silica, this may cause divergence of the beam after it refract at the internal surface of the micro-tube, which is the silica/test liquid interface. On the other hand, the test fluid with RI higher than that of the silica helps in confining the beam better and increasing the *Q*-factor. This happens until a certain extent, as the interspacing between the fundamental and higher order modes decreases with increasing the RI. When the interspacing is not enough to separate different peaks, intermodal interference occurs, like what is observed in the case of *n_t_* = 1.8 of Figure 5; in which, a strong coupling between the main peak and the side peak appears in the spectral response, and hence the main peak is smaller and wider; which renders the field spots to be of less intensity as in Figure 6d. The best performance regarding high transmission at the main peak, well confinement of light, and quite high *Q*-factor, is obtained with the case *n_t_* = 1.6, which is larger than the refractive index of the tube material, but not too large to reduce the separation between modes much, causing their coupling; it is also away from the critical values of the stability conditions.

A better quantitative comparison for the *Q*-factors and the confinement distances between the different cases is indicated in Table 1, from which one can notice that the confinement distance is smaller as *n_t_* increases, which is predicted. However, for *Q*-factors, the trend is not the straight forward. The decreasing intermodal spacing between the main modes and the side ones causes interference between them, leading to reduction in the *Q*-factor, as most pronounced for *n_t_* = 1.8.

## 4. Materials and Methods

**Figure 8:**
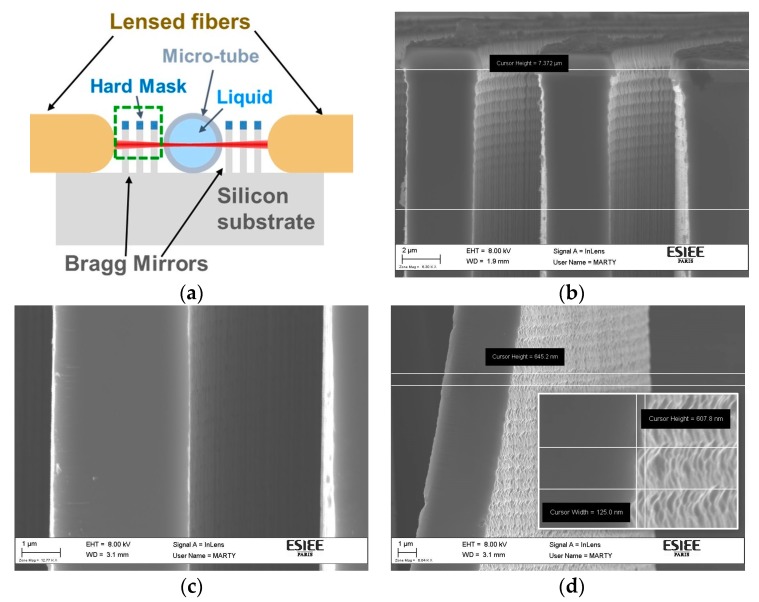
(**a**) Schematic diagram for the horizontal cross section of the device. (**b**) SEM image for the top part of one Bragg mirror indicated in schematic (a) by the dashed rectangle indicates the ARDSA phenomenon. (**c**) SEM image for the Bragg mirror at depth between 15 and 20 μm, corresponds to region illuminated by the light beams from the fiber, indicates the attenuatted scalloping. (**d**) SEM image for the outer surface of the Bragg mirror wall. The inset is a zoom indicating the dimensions of the non-attenuated scalloping.

Figure 8a shows a cross section schematic of the device. The implementation of the cavity is realized by Deep Reactive Ion Etching (DRIE) process on silicon substrate. The etching of two Bragg mirrors spaced by 280 μm is done after transferring the pattern onto a 400 nm-thick thermal oxide layer, as a hard mask, through a lithography step followed by fluorinated plasma etching of the oxide. Each Bragg mirror consists of three layers of silicon/air pair with thicknesses 3.67 μm and 3.49 μm respectively; both thicknesses correspond to odd multiple of quarter the central wavelength—which is 1550 nm—in the two mediums. The channel depths are 80 μm measured by our 3D profile-meter. Figure 8b shows a Scanning Electron Microscope (SEM) image for the top part of one Bragg mirror that is indicated in the schematic by the dashed rectangle. It can be noticed that the scalloping effect associated with the DRIE process is noticeable only within less than 7 μm depth from the silicon surface, then the scalloping attenuates in deep regions due to the phenomenon known as Aspect Ratio Dependent Scalloping Attenuation (ARDSA) [21]. This phenomenon appears in narrow openings with high aspect ratios, and is attributed to the transportation limit of radicals [21]. The important depth to us that is illuminated by the light, is estimated between 15 and 20 μm. The SEM image in Figure 8c shows that this region is scallop-free within the intermediate walls, which is very beneficial to reduce the scattering light loss. Nevertheless, the outward surfaces of the first and last mirrors suffer from surface roughness as they are not confined by narrow trenches. Figure 8d shows noticeable scalloping with undercut length and etched depth per cycle estimated by 125 and 645 nm, respectively. The mentioned undercut value render the root mean square roughness (σ) for these surfaces equal to about 44 nm. This value can be considered much less than the used light wavelength; besides, only two surfaces from five have this value while σ is equal to almost zero for the other three surfaces. Thus we believe the scattering losses can be negligible in our case. The mirror verticality has been measured to deviating from the exact perpendicular angle by about 2° only, which has a negligible effect on the mirror performance. The pronounced effect was due to the wall thickness change as for slight fabrication error. Several measurements has be done for silicon walls thickness and the air gabs in-between from the SEM images. The most deviated measurements have been found to be 3.36 and 4.497 μm for the silicon walls and air gaps, respectively. That leads to a theoretically estimated maximum reduction in the mirror reflectivity from 99.74% at the designed dimensions to 88.56% at the measure ones.

After the silicon chip fabrication, the fused silica micro-capillary tube is inserted inside the cavity and connected to external tubing allowing for the liquid insertion. The interfaces of the micro-capillary are expected to introduce parasitic reflection loss due to refractive index change. Estimated by Fresnel formula, each interface with air causes about 3.3% reduction in the transmitted power. The inner interface of the tube will introduce some losses also, depending on the refractive index of the passing liquid, but it is expected to be even smaller as the refractive index difference will be smaller in case of a liquid than that with air. Different mixtures of toluene and acetone are used to perform the test since the absorption of both being almost the same. To insure that, spectroscopy of pure toluene and that of different mixing ratios with acetone is performed using IR-Affinity-1 Fourier transform infrared spectrophotometer from Shimadzu connected in transmission mode.

The optical testing setup for the chip consists of a tunable laser source of model 81949A and a detector head with a power meter of model 81634B from Agilent (Santa Clara Valley, CA, USA), controlled using a computer. Injecting and collecting the light into and from the cavity is done using lensed fibers from Corning with typical spot size of 18 ± 2 μm and 300 μm working distance. Fiber positioners of five degrees of freedom are used to align the fibers inside the input and output grooves on the chip.

## 5. Results and Discussion

**Figure 9:**
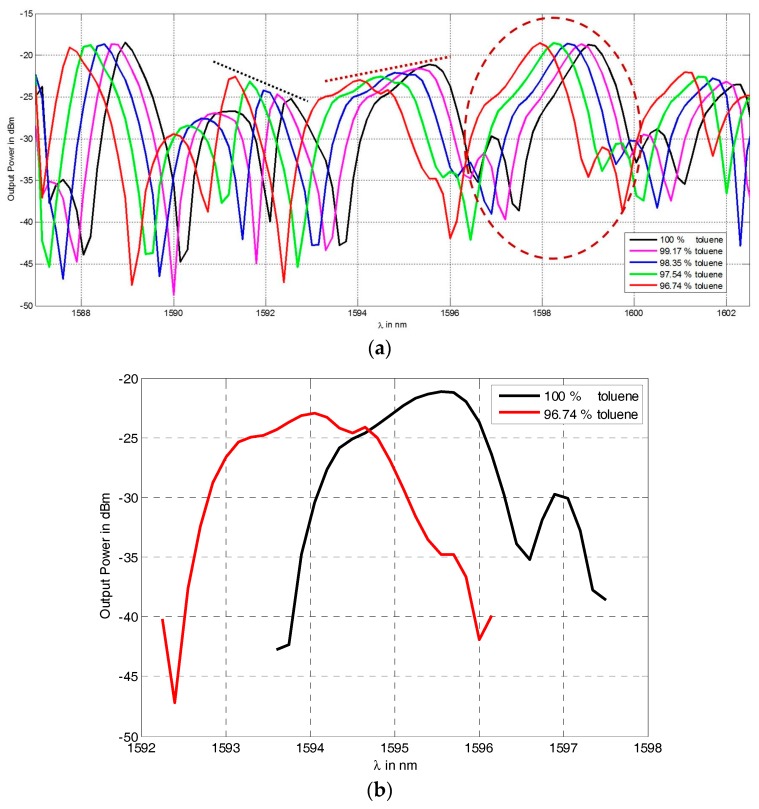
(**a**) The spectra of different mixture ratios of toluene and acetone measured by the proposed refractometry device. The peaks surrounded by the red dashed circle have the same maximum power transmission values, despite the different mixing ratios of toluene and acetone. (**b**) Zoom around the wavelength 1595 nm of the spectra of the two extreme mixing cases to indicate that the decrease in the transmitted power level upon changing the RI is due to the modal interference between the main peak and that of the higher order mode.

The transmission from the cavity is recorded while filling the capillary by mixtures of toluene and acetone of different volumetric ratios. Figure 9 plots these transmission spectra simultaneously to allow comparison. As inherited from Figure 9a, the peaks between the wavelengths of 1588 nm and 1600 nm (surrounded by the circle) have the same power transmission values, despite the different mixing ratios of toluene and acetone. The discrepancy between the maximal power values of the different curves at this peak is found to be less than 0.53 μW, which may be attributed to slight temperature changes, laser power instability, or alignment disturbance upon changing the liquids and running the scan.

From Figure 9a, it can be noticed that the peaks do not all behave in the same way, some have decreasing levels upon increasing the concentration of toluene like those around the wavelength 1592 nm (indicated by the tangent dashed black line with negative slope); other peaks have decreasing levels like those between the wavelengths of 1594 and 1596 nm (indicated by the tangent dotted red line with positive slope). To investigate the reason behind that, a close look is needed at the two extreme curves (100% and 96.74% concentration of toluene), which are magnified around the wavelength 1595 nm in Figure 9b. One notice that the side peak of the higher order mode in the black curve (at wavelength of 1597 nm for the 100% toluene) is merged with the main peak for the red curve of the lower toluene concentration as the RI changes. This is apparently the reason behind reducing the transmission level of the later spectrum. Note that, similar behavior was numerically demonstrated with HFSS simulations in Section 3 as the refractive index test liquid changes. Note also that the *Q*-factor for the main peaks free from modal interference—such as the first peaks between 1587 and 1590 nm in Figure 9a—is the highest and can reach up to 2896, while it is less in other peaks due to coupling with the higher order resonance peaks, similar to what has been observed with numerical modeling.

**Figure 10:**
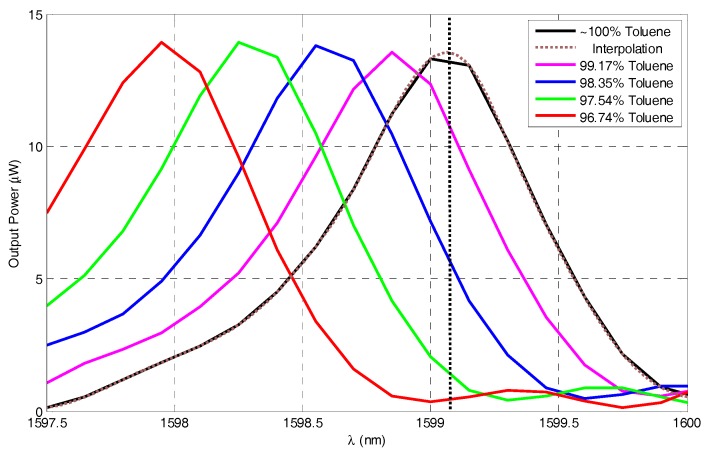
Zooming of the output power in μW *versus* wavelength in nm around the selected peak for refractometry analysis.

To characterize the performance of the refractometer, Figure 10 shows the zoomed view of the output power in μW *versus* wavelength in nm, which gives better linearity, around the selected peak. A reference line from the peak of a fitting curve of the pure toluene spectrum is used to trace the power drop upon the spectrum shift with the liquid RI changing. The error in wavelength between the measured peak and an interpolation is found to be less than 0.7 nm for all the curves and it is due to the poor measurement wavelength step of 1.5 nm.

Due to the resonance peak shifting upon RI change, the power drops along the reference line at this wavelength. Hence, the refractometer can be performing by tracing the power value only at a single wavelength after calibrating the system only once, but the range of detection by this method is limited by the range at which the side of the resonance peaks are almost linear, so the last curve is excluded as it cuts the reference line outside the linearity region.

**Figure 11:**
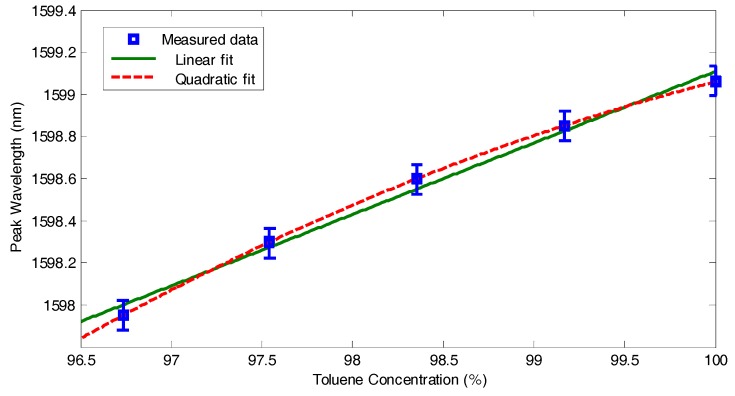
The position of the maxima wavelength *versus* the toluene concentration in the toluene-acetone mixture.

The common measurement technique by tracing the peaks′ wavelength maxima is also performed. The wavelengths shift is plotted *versus* the toluene concentration in Figure 11. As the measurement wavelength step is 0.15 nm, the actual maxima wavelength may be allocated within error of ±0.075 nm from the depicted values; this margin is indicated by the error bars in Figure 11. Note that the resulting relation is more like quadratic rather than linear, which indicates that the RI property for these liquids mixtures is not linearly additive upon volumetric ratios; hence it cannot be estimated from the known RI values of pure acetone and pure toluene. Similar nonlinearity has been observed for different liquid mixtures, which is thought to be caused be volume change upon mixing [22].

For the conventional method of tracing the peaks′ wavelength shift, the sensitivity $\delta \lambda /\delta n_{t}$ is analytically deduced to be:

(6) $$\frac{\delta \lambda }{\delta n_{t}}=\lambda \frac{d_{t}}{d}$$

The calculated sensitivity from the former equation gives a value of 428 nm/RIU. The allowed sensing range before interfering with the neighbor resonance peak (equivalent to the free spectral range of the resonator) is 3.45 nm, which is equivalent to about 0.008 RIU change in the test liquid RI. In can be noticed from Equation (6) that the ratio between the length containing the test liquid (the inner diameter of the microtube in our case) to the total cavity length (*d_t_/d*) is best to be 1 (or the closest to 1) for having the highest sensitivity. In our case *d_t_/d* = 75 μm/280 μm = 26.8% only. This renders the obtained sensitivity in our case lower than some other Fabry-Pérot cavities even with straight mirrors [15,23,24], but with the use the power drop technique rather than the conventional method of detecting the peak′s wavelength shift only, superior sensitivities can be attained as detailed hereafter. This could be due to the high *Q*-factor that exceeded 2800, which is the highest value reported for an on-chip Fabry-Pérot refractometer.

**Figure 12:**
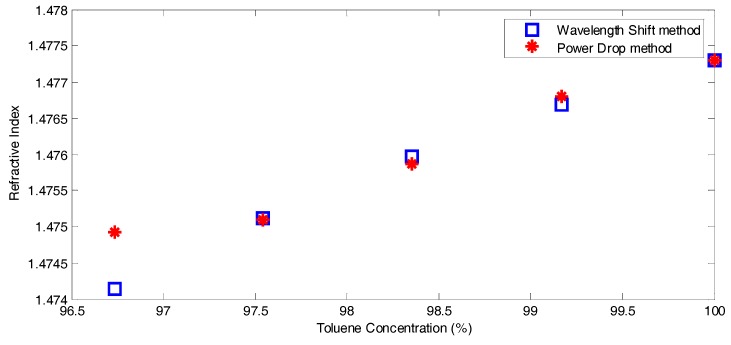
The estimated refractive index *versus* the toluene concentration in the toluene-acetone mixture.

The calculated RI of the unknown mixture obtained by the wavelength shift method is employed to calibrate the sensor operating in the mode of tracing the power drop, to get its sensitivity$\;\delta P/\delta n_{t}$. Figure 12 shows the obtained RI values *versus* Toluene concentration, a good agreement between both methods is obtained at $\delta P/\delta n_{t}$ of approximately 5500 μW/RIU. The range in this case is −2.73 μW < Δ*P* < −12.12 μW, that is equivalent to 0.0005 < Δ*n* < 0.0022. Note that the last point is far from the linear region, and hence it does not fit with the expected RI value.

## 6. Conclusions

Designing a stable Fabry–Pérot cavity employing in-plan cylindrical mirrors and out-of-plan curved surface of a micro-tube has been detailed in this article. By the well confinement of the light inside the cavity attained by the proper design, interference spectrum with high *Q*-factor resonance peaks can be achieved. Such narrow spectral peaks with fast roll-off are useful for sensing applications. On the other hand, such resonator with curved surfaces exhibits higher order resonance modes which may interfere with the principle resonance peak lowering the *Q*-factor as investigated by numerical simulations and observed experimentally. A proper choice of the resonance peak is then critical to achieve high performance sensor. The experimental testing using mixtures of toluene and acetone has been done. By tracing the peak maxima shift in wavelength upon changing the analyte RI, sensitivity up to 428 nm/RIU is achieved along a range of 3.45 nm. High sensitivity up to 5500 μW/RIU can be reached by employing the technique of power tracing at a fixed wavelength, but on a limited range of 0.0005 < Δ*n* < 0.0022. The later method has the advantage of working at a single wavelength and requiring only an optical detector, without the need for sophisticated spectrometry equipment after the device calibration. Noting that, this technique is useful only when absorption difference between the analytes is not significant.

## Figures and Tables

**Table 1:** Comparison between the *Q*-factor and the confinement distance between different test liquid filling the tube.

RI of Test Liquid	*n_t_* = 1.18	*n_t_* = 1.3	*n_t_* = 1.6	*n_t_* = 1.8
*Q_peak_*	70	129.5	128	55
Confinement distance	1.88 μm	1.34 μm	1.24 μm	1.11 μm

## References

[1] Liu P.Y., Chin L.K., Ser W., Chen H.F., Hsieh C.-M., Lee C.-H., Sung K.-B., Ayi T.C., Yap P.H., Liedberg B. (2016). Cell refractive index for cell biology and disease diagnosis: Past, present and future. Lab Chip.

[2] Shelton D.P. (2011). Refractive index measured by laser beam displacement at λ = 1064 nm for solvents and deuterated solvents. Appl. Opt..

[3] Chaitavon K., Sumriddetchkajorn S., Nukeaw J. (2013). Highly sensitive refractive index measurement with a sandwiched single-flow-channel crofluidic chip. RSC Adv..

[4] Chao C.-Y., Fung W., Guo L.J. (2006). Polymer microring resonators for biochemical sensing applications. IEEE J. Sel. Top. Quantum Electron..

[5] Gaber N., Takemura Y., Marty F., Khalil D., Angelescu D., Richalot E., Bourouina T. (2015). Volume refractometry of liquids using stable optofluidic Fabry-Pérot resonator with curved surfaces. J. Micro/Nanolithgr. MEMS MOEMS.

[6] Homola J., Yee S.S., Gauglitz G. (1999). Surface plasmon resonance sensors: Review. Sens. Actuators B Chem..

[7] Schmitt K., Schirmer B., Hoffmann C., Brandenburg A., Meyrueis P. (2007). Interferometric biosensor based on planar optical waveguide sensor chips for label-free detection of surface bound bioreactions. Biosens. Bioelectron..

[8] Madani A., Kleinert M., Stolarek D., Zimmermann L., Ma L., Schmidt O.G. (2015). Vertical optical ring resonators fully integrated with nanophotonic waveguides on silicon-on-insulator substrates. Opt. Lett..

[9] Bernardi A., Kiravittaya S., Rastelli A., Songmuang R., Thurmer D.J., Benyoucef M., Schmidt O.G. (2008). On-chip Si/SiO*x* microtube refractometer. Appl. Phys. Lett..

[10] Böttner S., Li S., Trommer J., Kiravittaya S., Schmidt O.G. (2012). Sharp whispering-gallery modes in rolled-up vertical SiO_2_ microcavities with quality factors exceeding 5000. Opt. Lett..

[11] Harazim S.M., Quiñones V.A.B., Kiravittaya S., Sanchez S., Schmidt O.G. (2012). Lab-in-a-tube: On-chip integration of glass optofluidic ring resonators for label-free sensing applications. Lab Chip.

[12] Zhang X., Ren L., Wu X., Li H., Liu L., Xu L. (2011). Coupled optofluidic ring laser for ultrahigh sensitive sensing. Opt. Express.

[13] Domachuk P., Littler I., Cronin-Golomb M., Eggleton B. (2006). Compact Resonant Integrated Microfluidic Refractometer. Appl. Phys. Lett..

[14] Song W., Zhang X., Liu A., Lim C., Yap P., Hosseini H. (2006). Refractive index measurement of single living cells using on-chip Fabry-Pérot cavity. Appl. Phys. Lett..

[15] St-Gelais R., Masson J., Peter Y.-A. (2009). All-silicon integrated Fabry-Pérot cavity for volume refractive index measurement in microfluidic systems. Appl. Phys. Lett..

[16] Sabry Y.M., Saadany B., Khalil D., Bourouina T. (2013). Silicon micromirrors with three-dimensional curvature enabling lens-less efficient coupling of free-space light. Light Sci. Appl..

[17] Sabry Y.M., Khalil D., Saadany B., Bourouina T. (2014). In-plane external fiber Fabry-Pérot cavity comprising silicon micromachined concave mirror. J. Micro/Nanolithgr. MEMS MOEMS.

[18] Saleh B.E.A., Teich M.C. (1991). Fundamentals of Photonics.

[19] Yariv A. (1989). Quantum Electronics.

[20] Malak M., Gaber N., Marty F., Pavy N., Richalot E., Bourouina T. (2013). Analysis of Fabry-Pérot optical micro-cavities based on coating-free all-Silicon cylindrical Bragg reflectors. Opt. Express.

[21] Mita Y., Sugiyama M., Kubota M., Marty F., Bourouina T., Shibata T. Aspect Ratio Dependent Scalloping Attenuation in DRIE and an Application to Low-Loss Fiber-Optical Switches. Proceedings of the 19th IEEE International Conference on Micro Electro Mechanical Systems (MEMS 2006).

[22] Kurtz S.S., Wikingsson A.E., Camin D.L., Thompson A.R. (1965). Refractive Index and Density of Acetone-Water Solutions. J. Chem. Eng. Data.

[23] Wei T., Han Y., Li Y., Tsai H.-L., Xiao H. (2008). Temperature-insensitive miniaturized fiber inline Fabry-Pérot interferometer for highly sensitive refractive index measurement. Opt. Express.

[24] Liu P., Huang H., Cao T., Tang Z., Liu X., Qi Z., Ren M., Wu H. (2012). An optofluidics biosensor consisted of high-finesse Fabry-Pérot resonator and micro-fluidic channel. Appl. Phys. Lett..

